# Leaching of PCBs and Nutrients from Soil Fertilized with Municipal Sewage Sludge

**DOI:** 10.1007/s00128-016-1802-y

**Published:** 2016-04-23

**Authors:** Magdalena Urbaniak, Ilona Gągała, Mariusz Szewczyk, Agnieszka Bednarek

**Affiliations:** Department of Applied Ecology, Faculty of Biology and Environmental Protection, University of Lodz, Banacha 12/16, 90-237 Lodz, Poland; European Regional Centre for Ecohydrology of the Polish Academy of Sciences, Tylna 3, 90-364 Lodz, Poland

**Keywords:** Sewage sludge, Leachate, PCBs, Nutrients

## Abstract

Although sewage sludge is a rich source of nutrients for arable farming and soil improvement, it can also be a source of pollutants. The effects of the land application of sludge on the PCB and nutrient content of leachate were investigated using cylindrical 650 mm length columns filled with poor quality soil. Treatments included no fertilization (control), fertilization using a 62.5 t/ha dose (O50) of sewage sludge from the largest Polish Wastewater Treatment Plant, in Lodz, and a 62.5 t/ha dose of sewage sludge mixed with CaO (O50Ca). The leaching of sludge-borne PCBs and nutrients was simulated by the application of distilled water in a quantity reflecting the annual rainfall of 562.5 mm. The obtained results demonstrate that application of sewage sludge and water simulated leaching of the most mobile chemical compounds, nitrate for example, whereas the addition of CaO decreased the average PCB and phosphorus concentrations in comparison to the control and O50 samples.

**Electronic supplementary material:**

The online version of this article (doi:10.1007/s00128-016-1802-y) contains supplementary material, which is available to authorized users.

Recent years have seen increasing interest in the pathways of pollutant migration in soils fertilized with sewage sludge (Wilson et al. 1997; Molina et al. 2000; Hesselsoe et al. 2001). The application of sewage sludge to land is widespread, as it represents a valuable source of nutrients, particularly nitrogen and phosphorus, and organic matter for arable farming and reconditioning of sandy and degraded soils (McLachlan et al. 1996; Łuczkiewicz 2006). Although sewage sludge improves soil properties, numerous studies have demonstrated that they also contain toxic organic compounds, such as polychlorinated biphenyls (PCBs) (McLachlan et al. 1996; Urbaniak et al. 2014; Wyrwicka et al. 2014), which have mutagenic, carcinogenic, immunotoxic as well as developmental and reproductive effects in living organisms (Urbaniak 2007). Despite the fact that PCBs show a strong affinity to organic matter which predispose them to be stored in the sediments (Gdaniec-Pietryka et al. 2013) and surface layer of the soil, it has been also shown that they can be transferred deeper into the soil profile (Bi et al. 2002; Kobasić et al. 2008; Zhang et al. 2011). Hence, the continued use of such contaminated sludge for agricultural purposes can present problems associated with the risk of soil, subsurface and groundwater contamination (Bi et al. 2002). This also concerns nutrients, which despite being valuable from the perspective of agriculture, their combined application with sewage sludge may lead to health and environmental risks associated with subsurface and groundwater pollution (Kang et al. 2011). For example, as nitrates and nitrites are not retained in soils, they quickly partition to any water phase, and if not assimilated by plants, will enter surface waters through runoff. This in turn, together with phosphate leaching (Ulen and Etana, 2010; Kang et al. 2011), contributes to the acceleration of eutrophication in water reservoirs, causing deterioration of water quality and the promotion of toxic water blooms dominated by cyanobacteria (Sharpley et al. 2003; Boesch et al. 2005; Gągała et al. 2014).

Hence, the assessment of groundwater contamination as a result of the application of sewage sludge is an extremely important element for management of soil and groundwater resources. This paper studies the effects of land application of sewage sludge in column experiments with the aim of determining the ability of the sludge-borne PCBs and nutrients to leach from amended soil. Enzyme-linked immunosorbent assay’s (ELISA) were used which allowed rapid and cost-effective analysis of PCBs in comparison to the traditional analytical approach.

## Materials and Methods

Sewage sludge from the biggest Municipal Wastewater Treatment Plant in Poland located in Lodz was collected and used as fertilizer for the poor quality soil (class VI, according to the Regulation of the Council of Ministers of 12 September 2012 on the soil science and classification of land; OJ 2012 item, 1246). The used sludge were contaminated by heavy metals and organic pollutants such as dioxins and PCBs as was demonstrated in our earlier study by Urbaniak et al. (2014) and Wyrwicka et al. (2014).

The cylindrical PVC columns were used for the evaluation of PCB, total nitrogen (TN) and total phosphorus (TP) and ion (NO_3_^−^, NO_2_^−^, NH_4_^+^, PO_4_^3−^) concentrations in leachate. The length of each column was 650 mm with an internal diameter of 100 mm. The column experiment was conducted in 3 triplicate variants: (1) only soil (control); (2) soil fertilized with 62.5 t/ha dry weight (d.w.) sewage sludge (O50); (3) soil fertilized with 62.5 t/ha d.w. sewage sludge mixed with 40 % CaO d.w. (O50Ca). The experiment continued for 15 days.

Leachate samples for the analysis of PCBs, TN, TP and ions (NO_3_^−^, NO_2_^−^, NH_4_^+^, PO_4_^3−^) were collected daily, starting 24 h after the initial application of 300 mL of distilled water. This procedure was repeated daily. A dose of 300 mL of water applied for 15 days was calculated to reflect the annual rainfall of 562.5 mm, which is closest to the typical annual rainfall for the Lodz region (Central Poland) amounting to 572 mm.

The PCB content of the leachate samples was analyzed using a PCB Rapid Assay kit, based on method described earlier by Wyrwicka et al. (2014). TP was analyzed by the ascorbic acid method (Greenberg et al. 1992), following digestion by Oxisolv (Merck), an oxidizing decomposition reagent, with the MV 500 Microwave Digestion System (Merck). TN was analyzed using the persulphate digestion method (HACH, 1997). The ions (NO_3_^−^, NO_2_^−^, NH_4_^+^, PO_4_^3−^) were analyzed by using an ion chromatograph (Dionex Corporation, ICS-1000) according to methods described earlier by Urbaniak et al. (2012) and Gągała et al. (2014).

## Results and Discussion

With regard to PCB concentrations in groundwater only a few studies have been dedicated to this issue due to general low solubility of PCBs in water and hence, their strong tendency to be sorbed onto soil (Adeel et al. 1997; Kobasić et al. 2008). General conclusions regarding their presence in groundwater are based solely on the gross behavior of the Aroclor mixtures, and do not consider the fact that they represent a complex mix of a large number of PCB compounds that vary with regard to their solubility in water and potential for sorption and biodegradation. For example, on the basis of water solubility and *n*-octanol–water partition coefficients, the lower chlorinated congeners are not sorbed as strongly as the higher chlorinated isomers, and thus tend to leach more readily. Thus, lower chlorinated PCBs tend to have a greater presence in groundwater (Bi et al. 2002).

**Fig. 1:**
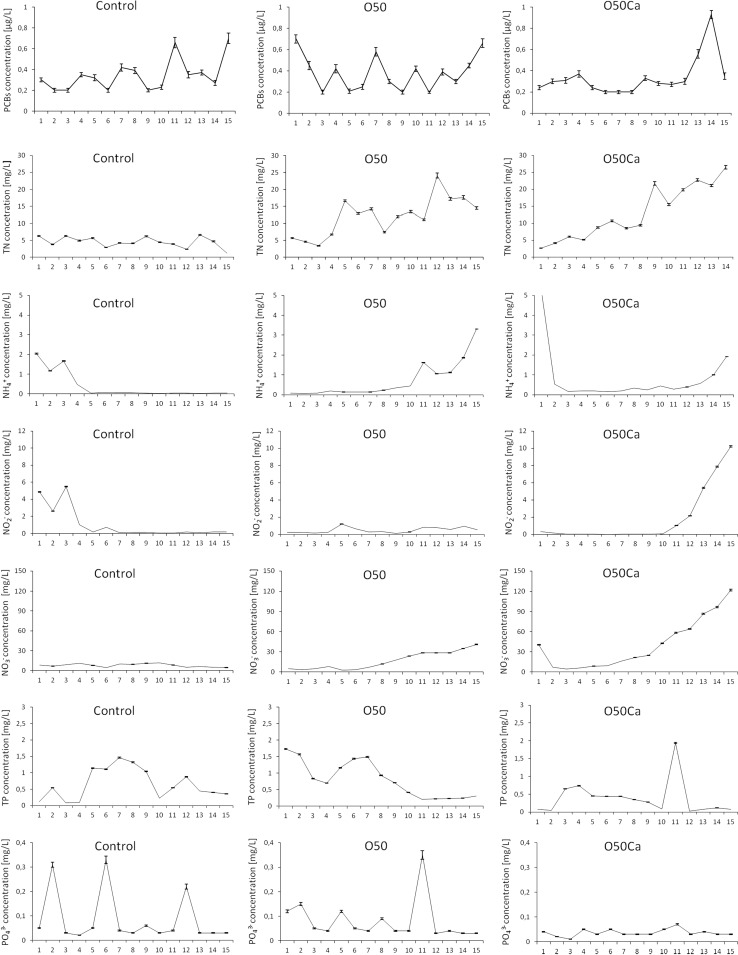
The PCBs and nutrients concentrations in the leachate samples from control soil (control), soil amended with sewage sludge (O50) and mixture of sewage sludge and CaO (O50Ca) during 15-days experiment

In the present study, PCB analysis revealed a slight increase of the average concentration from 0.40 ± 0.15 µg/L in the control to 0.43 ± 0.15 µg/L in leachate collected from soil treated with sewage sludge. The leachate from soil fertilized with the mixture of sewage sludge and CaO demonstrated a decrease in average PCB concentration in comparison to both the control and O50 samples; however, the obtained differences were not statistically relevant (*p* ≥ 0.05). With regard to PCB concentrations observed at individual days, the values varied between not detectable (below 0.20 µg/L) to 0.70 µg/L in the case of the control and O50 samples, while higher concentrations were noted in the case of leachate collected from the O50Ca samples (between not detected to 0.93 µg/L; Fig. 1). Despite this, the average PCB concentration in the O50Ca leachate samples was the lowest of all the variants (0.37 ± 0.20 µg/L).

The total amount of PCBs eluted with the applied volume of water is presented in Table 1. The lowest amount was noted for the O50Ca (1.42 ± 0.09 µg), indicating that CaO plays a role in the retention of PCBs in the soil profile, whereas the highest amount was noted for O50 (1.54 ± 0.13 µg), due to presence of sludge-borne PCBs. The used dose of PCBs with the sludge amounted to 12.30 ± 0.43 µg. Thus the leaching of PCBs was minimal (10.89 %, 12.20 % and 11.54 % in the case of control, O50 and O50Ca), as these compounds exhibit a hydrophobic character and thus are not readily transported with water: the majority being retained in the soil profile. These findings confirm those of a study of the leaching of PCBs from soil containing sludge and soil contaminated with hydraulic oil into groundwater by Adeel et al. (1997), which demonstrated that only a small portion of sludge-born PCB (less than 6 %) was removed from the soil by prolonged leaching.

**Table 1:** The total amount of eluted PCBs and nutrients from control soil (control), soil amended with sewage sludge (O50) and mixture of sewage sludge and CaO (O50Ca) during 15-days experiment

Compound Unit	PCB µg	TN mg	NH_4_^+^ mg	NO_2_^−^ mg	NO_3_^−^ mg	TP mg	PO_4_^3−^ mg
Control	1.34 ± 0.12	20.90 ± 0.56	1.77 ± 0.02	4.83 ± 0.05	36.31 ± 0.36	3.05 ± 0.03	0.41 ± 0.02
O50	1.54 ± 0.13	60.09 ± 1.43	3.69 ± 0.03	2.39 ± 0.02	83.27 ± 0.76	3.56 ± 0.04	0.38 ± 0.02
O50Ca	1.42 ± 0.09	72.01 ± 1.31	2.30 ± 0.03	8.84 ± 0.08	189.04 ± 1.87	1.92 ± 0.02	0.17 ± 0.01

In turn, Kobasić et al. (2008) report much lower leaching (0.34 %) of the sum of seven indicator PCBs around a damaged capacitor, with the majority of the PCB congeners washed away in the summer due to higher temperatures and heavier rainfall. Despite this, the authors indicate the presence of much higher PCB concentrations than those obtained in the present study, ranging from 1.60 to 22.25 µg/L. The differences in the obtained portions of leached PCBs could mostly be attributed to the fact that Kobasić et al. (2008) analyzed only seven indicator congeners, whereas the present study addresses total PCB concentration (Σ 209 PCB congeners), including a variety of congeners of diverse solubility. In consequence, the higher rate of eluted PCBs observed in our case may be attributed to the lower chlorinated and thus less hydrophobic congeners. In addition, the higher concentrations of PCBs observed by Kobasić et al. (2008) could be attributed to their choice of analytical technology, i.e. instrumental chromatographic analysis, as the present study is based on an ELISA test kit: some ELISA readings can be underestimated in comparison to conventional chromatographic analysis (Nording et al. 2006; Tsutsumi et al. 2006) resulting in the higher concentration noted by the authors. Kobasić et al. (2008) collected leachate samples over a 1-year period following precipitation events, and documented no accelerated or time-restricted washing of PCBs from soil profiles similar to the present study.

The relatively similar concentrations (Fig. 1) and amounts of eluted PCBs (Table 1) observed in the control, O50 and O50Ca samples may be the effect of releasing some portions of PCBs from the used cylindrical PVC columns. As it is reported in the UNEP guidelines (UNEP, 1999) PVC materials act as source PCBs in the environment. The obtained results also demonstrate the role of CaO in diminishing PCB leaching from soil fertilized with sewage sludge. This can be an effect of PCB dechlorination in the presence of CaO as it was demonstrated by Mitoma et al. (2013).

The differences in the obtained results can be also related to the permeability, porosity, homogeneity, texture, and mineralogy of the soil, which affect the desorbability of PCBs. Haque and Schmedding (1976) report that PCB sorption in different sorbents (sand, soil, clay and humic acids) occurs in order of reducing chlorination, i.e. hexa- > tetra- > dichlorobiphenyls. Other studies note that sorption of PCB in soil increases with increasing soil organic carbon content (Urbaniak 2007; Zhang et al. 2011). In consequence, as the sorption reaction influences the transport and fate of PCBs and their partitioning in different compartments of the soil-groundwater system, a simple comparison of our findings with other data is not possible.

In the case of TN, the maximum values were noted in leachate eluted from soil fertilized with the sewage sludge mixed with CaO (up to 36.50 mg/L). The same dose used without CaO caused lower TN concentrations (maximum 24.10 mg/L; Fig. 1). Similar results were obtained in the case of the total amount of eluted TN (Fig. 1). Therefore significant differences in TN concentration between the control and O50, and between the control and O50Ca leachate samples were noted (*p* ≥ 0.05), while there were no statistically relevant differences observed in the case of O50 and O50Ca (*p* ≥ 0.05). These values are higher than those noted in previous studies. For example, Duan et al. (2010) report the concentration of TN in leachate from a wastewater land application system to be significantly lower than 10 mg/L, therefore demonstrating no potential nitrogen contamination for groundwater. In our case, the concentration of TN exceeded 10 mg/L in 10 and 8 samples of O50 and O50Ca, respectively, with the higher average value noted for O50Ca (Fig. 1). In contrast, concentrations above 10 mg/L were not observed for the control samples.

With regard to the nitrogen ions, the obtained results demonstrated the enhanced concentrations of nitrate, reaching maximum values of 11.30, 40.94 and 122.03 mg/L in control, O50 and O50Ca samples, respectively; whereas nitrite and ammonium demonstrated values which were several times lower (Fig. 1). Furthermore, all the ions showed different concentrations over time: ammonium demonstrated increased values at the beginning of the experiment in the control samples, while in O50 and O50Ca, the highest concentrations occurred at the end of the experiment. The exception was the O50Ca sample, in which the highest noted ammonium concentration (5.27 mg/L) occured on Day 1 of the experiment. Nitrite concentrations showed a similar trend to ammonium, with elevated concentrations noted for O50Ca samples at the end of the experiment. O50 samples demonstrated the highest values one-third-way through the experiment, i.e. on the 5th and 6th days, and then at the end of the test period, whereas the control samples revealed the greatest values at the beginning of the experiment (Fig. 1). In turn, fluctual changes were observed in nitrate concentration, with elevated values noted at the beginning, until the 4th day, and in the middle of the experiment, on the 9th and 10th days, with lower concentrations interspersed between them (Fig. 1). Leachate from O50 showed increased nitrite values, above 10 mg/L, starting from the 8th day of the experiment. However, leachate collected from O50Ca, showed high concentrations at the 1st day (40.38 mg/L), followed by a rapid decline (below the 10 mg/L) during the next 5 days, and then increasing again from the 7th day, with concentrations ranging from 16.41 up to 122.03 mg/L on the last day (Fig. 1).

In relation to the obtained high concentrations of nitrate, it needs to be emphasized that this form of nitrogen is the most mobile in soil and hence the most problematic potential pollutant of waters, being associated with e.g. methemoglobinemia and carcinogenic and mutagenic changes observed mainly in the digestive system. Regarding these negative health effects, the World Health Organization has set the limit for nitrate in potable water at 50 mg/L (Drinking Water Directive 98/83/EC). The same limit value was also established for groundwater (Nitrates Directive 91/676/EEC), while EPA’s sets the maximum contaminant level for nitrate at lower level of 10 mg/L. The obtained results exceed these allowable limits of 50 mg/L only in the case of O50Ca reaching 122.03 mg/L at the highest, while 10 mg/L limit was exceeded also in the case of O50 reaching 40.94 mg/L at the highest (Fig. 1). The high mobility of nitrate is also observed in the amount of eluted compounds, which reached 36.31 ± 0.36, 83.27 ± 0.76 and 189.04 ± 1.87 mg in control, O50 and O50Ca, respectively, whereas nitrite and ammonium achieved maximum values of 8.84 ± 0.08 (O50Ca) and 3.69 ± 0.03 mg (O50; Table 1). This is a clear indication of the problem associated with applying sewage sludge to fields, resulting in enhanced nitrate leaching and a greater risk to groundwater.

In the case of phosphorus, the present study showed that application of sewage sludge at a dose of 62.5 t/ha resulted in a 25 % increase in average TP concentration, whereas application of mixture of sewage sludge and CaO resulted in a 40 % decrease of average TP concentration (Fig. 1). Similarly, while the highest total amount of the washed TP was demonstrated in the case of O50, this value observed in leachate from the soil amended with sewage sludge and CaO was 1.6 times lower than that of the control soil, however the obtained differences were not statistically different (*p* ≥ 0.05) (Fig. 1). Also, in the case of orthophosphate, CaO was demonstrated to play a decreasing role (Fig. 1; Table 1). The Ca in soil reacts with phosphate and leads to the formation of dibasic calcium phosphate dihydrate, octocalcium phosphate, and hydroxyapatite. The formation of each product results in a decrease in the solubility and availability of orthophosphate, thus diminishing its concentration in the soil leachate samples amended with the mixture of sewage sludge and CaO. Hence, the application of such mixtures seems to be an effective way to minimize the risk related to sewage sludge usage.

To conclude, our findings show that the application of sewage sludge and water increase leaching of nitrate, as well as other highly mobile chemical compounds, with the highest rate observed in the case of sewage sludge and CaO mixture. On the other hand, compounds with high affinity to organic matter such as PCBs, TP and orthophosphate, decreased as an effect of CaO addition, demonstrate the potential of the use of CaO as a means of reducing the risk of the presence of the above compounds in groundwater.

## Electronic supplementary material

Below is the link to the electronic supplementary material.

Supplementary material 1 (DOCX 17 kb)
