# Supplementary material for: Leaching of PCBs and Nutrients from Soil Fertilized with Municipal Sewage Sludge
Source: Bull Environ Contam Toxicol. 2016 Apr 23;97:249–54. doi: 10.1007/s00128-016-1802-y (PMC4942498; doi:10.1007/s00128-016-1802-y)
Supplement: Supplementary file 1 — Supplementary material 1 (DOCX 17 kb) [file 128_2016_1802_MOESM1_ESM.docx]

**Supplementary materials**

**PCB analysis**

The PCB content of the leachate samples was analyzed using a PCB Rapid Assay kit (A00133/A00134) purchased from Tigret Sp z.o.o., based on producer guidance. An aliquot (200 mL) of calibration standard PCB delivered together with the assay kit of known concentrations: 0.00; 0.25; 1.00 and 5.00 µg/L (A00133/A00134), the positive control solution of 3.00 µg/L also delivered together with the assay kit and (A00133/A00134) the leachate sample were added to test tubes together with aliquots of enzyme conjugate (250 mL). After this, an aliquot (500 mL) of antibody, coupled with magnetic particles in buffered saline containing preservative and stabilizers, was added, thoroughly mixed and incubated at room temperature for 15 min using a RaPID Magnetic Separator. After incubation, the contents of each vial were decanted to a waste container to remove the solution containing any unbound reagents. The vials were then washed twice with washing solution (1 mL/vial). Following washing, an aliquot (500 mL) of color solution containing hydrogen peroxide and 3,3’,5,5’-tetramethylobenzidine in an organic base was added to each vial, shaken and incubated for 20 min. At the end of the incubation period, an aliquot (500 mL) of stopping solution, containing 2 M sulphuric acid was added to each vial. The absorbance of the liquid in each vial was measured at 450 nm using an SDI Differential Spectrophotometer.

**Nutrient analysis**

Total forms of nutrient were analyzed in unfiltered water. TP was analyzed by the ascorbic acid method (Greenberg et al. 1992), following digestion by Oxisolv (Merck), an oxidizing decomposition reagent, with the MV 500 Microwave Digestion System (Merck). TN was analyzed using the persulphate digestion method (HACH, 1997).

The ions (NO_3_^-^, NO_2_^-^, NH_4_^+^, PO_4_^3-^) were analyzed in filtered water samples by using an ion chromatograph, which consists of two ion chromatography systems separated for anions and cations (Dionex Corporation, ICS-1000). Each ion chromatograph consists of a pomp, eluent, guard column (CG18 for cations and AG22 for anions), an analytical column (IonPac CS18 for cation, IonPac AS22 for anion), and an electrolytic suppressor (CSRS-ULTRA II cation electrolytic suppressor and ASRS – ULTRA II anion electrolytic suppressor) to stabilise the baseline. The analysis was performed with 16 mM methanesulphonic acid (Fluka) for the cation analysis and a mixture of 4.5 mM sodium carbonate and 1.4 mM sodium bicarbonate for the anion system prepared from the AS22 Eluent Concentrate (Dionex Corporation). Both ion chromatographs were operated in isocratic elution in 30 ºC at a flow rate of 1 mL min^-1^. Measurements were performed using a 25 µL injection loop. For ion identification, combined standards were used (Seven Anion Standard II, Dionex Six Cation Standard produced by Dionex Corporation) (Urbaniak et al. 2012).

**QA/QC**

**PCBs**

Each analytical batch contained a sample blank, a control sample of known concentration (3 ng L^-1^, as Aroclor 1254), calibration standards and samples. The precision was verified by duplicate analyses and the test reproducibility was measured using coefficient of variation (CVs). The CVs should be lower than 10% for standard duplicates. If the CVs exceeded the above values the whole procedure was repeated in order to achieve good quality of the obtained results. The minimum method detection limit was 0.20 µg/L.

**Nutrients**

The samples for TP and TN were analyzed in duplicates. The reagent blank was used repeatedly for each measurement. In order to check the validity of the measurements, the calibration curve, using the standards of known TN and TP concentrations, was generated with R^2^=0.9997. The accuracy for TP was 5% and for TN 3%. The method detection limit for TN was 0.10mg/L and for TP was 0.03 mg/L.

In the case of ions, the samples were also analyzed in duplicates. The ions concentrations were calculated automatically by calibration curves prepared from standards. The accuracy for nitrites, nitrates, ammonium and phosphate was between 1%- 3% and the method detection limit was 1 μg/L and the limit of quantification was 10 μg/L.
